# High-content siRNA screening of the kinome identifies kinases involved in Alzheimer's disease-related tau hyperphosphorylation

**DOI:** 10.1186/1471-2164-11-25

**Published:** 2010-01-12

**Authors:** David O Azorsa, RiLee H Robeson, Danielle Frost, Bessie Meec hoovet, Gillian R Brautigam, Chad Dickey, Christian Beaudry, Gargi D Basu, David R Holz, Joseph A Hernandez, Kristen M Bisanz, Leslie Gwinn, Andrew Grover, Joseph Rogers, Eric M Reiman, Michael Hutton, Dietrich A Stephan, Spyro Mousses, Travis Dunckley

**Affiliations:** 1 Neuorgenomics Division, Translational Genomics Research Institute, Phoenix, Arizona 85004, USA; 2 Pharmaceutical Genomics Division, Translational Genomics Research Institute, Scottsdale, Arizona, 85251, USA; 3 Department of Neurology, Mayo Clinic, Jacksonville, FL, USA; 4 Center for Alzheimer's Research, Sun Health Research Institute, Sun City, Arizona, USA; 5 Banner Alzheimer's Institute and Department of Psychiatry, University of Arizona, Phoenix, AZ, USA; 6 Arizona Alzheimer's Consortium, Phoenix, AZ, USA; 7 Senior Director, Neuroscience Drug Discovery, Merck and Co Ltd., BMB8-106, 33 Avenue Louis Pasteur, Boston MA 02115, USA; 8 Department of Molecular Pharmacology and Physiology, College of Medicine, University of South Florida, 12901 Bruce B. Downs Blvd, MDC 8, Tampa, FL 33612, USA

## Abstract

**Background:**

Neurofibrillary tangles (NFT), a cardinal neuropathological feature of Alzheimer's disease (AD) that is highly correlated with synaptic loss and dementia severity, appear to be partly attributable to increased phosphorylation of the microtubule stabilizing protein tau at certain AD-related residues. Identifying the kinases involved in the pathologic phosphorylation of tau may provide targets at which to aim new AD-modifying treatments.

**Results:**

We report results from a screen of 572 kinases in the human genome for effects on tau hyperphosphorylation using a loss of function, high-throughput RNAi approach. We confirm effects of three kinases from this screen, the eukaryotic translation initiation factor 2 α kinase 2 (EIF2AK2), the dual-specificity tyrosine-(Y)-phosphorylation regulated kinase 1A (DYRK1A), and the A-kinase anchor protein 13 (AKAP13) on tau phosphorylation at the 12E8 epitope (serine 262/serine 356). We provide evidence that EIF2AK2 effects may result from effects on tau protein expression, whereas DYRK1A and AKAP13 are likely more specifically involved in tau phosphorylation pathways.

**Conclusions:**

These findings identify novel kinases that phosphorylate tau protein and provide a valuable reference data set describing the kinases involved in phosphorylating tau at an AD-relevant epitope.

## Background

Alzheimer's disease (AD) is a secondary tauopathy, generally thought to result from the upstream effects of toxic amyloid aggregates. While there are a growing number of amyloid-modifying therapeutics in clinical trials, targeting amyloid alone may not be sufficient to mitigate the cognitive deficits that occur during the full course of AD[1]. Other targets, such as the tau and apolipoprotein E proteins, are thus being investigating for additional therapeutic development. However, the proteins through which amyloid signals to promote tau pathology in AD are a critical missing link that must be connected to facilitate the development of tau-modifying AD treatments.

In AD, tau protein becomes hyperphosphorylated and aggregates into paired helical filaments (PHF), the main component of NFTs [2-7]. Indeed, altered tau protein function has emerged as a key factor in many neurodegenerative diseases, including AD[8,9]. Tau functions as a microtubule organizing protein that increases microtubule stability by suppressing dynamic instability[10]. Hyperphosphorylation of tau protein is thought to lead to microtubule instability, neurofibrillary tangle formation, and loss of a functional microtubule cytoskeleton, contributing to neuronal cell dysfunction and cell death. In Alzheimer's disease, sequential hyperphosphorylation of tau protein on multiple amino acids correlates with the severity of NFT pathology in affected brain regions[11]. There are numerous tau phosphorylation sites associated with tau dysfunction and neurodegeneration[12]. Phosphorylation of tau protein on serine 262 has been demonstrated to significantly reduce the affinity of tau protein for microtubules[13] and this serine is hyperphosphorylated early in progression of disease pathology, before mature NFTs form[11]. Thus, increased ser262 phosphorylation is an important initial step in the pathological progression to cytoskeletal dysfunction and NFT formation in Alzheimer's disease, although full neurodegenerative effects likely require hyperphosphorylation of multiple sites[14]. Identifying the kinases involved in ser262 hyperphosphorylation will increase our understanding of the mechanisms causing tau and cytoskeletal dysfunction in AD, and could provide new targets for the discovery of tau-modifying AD treatments.

*In vitro*, numerous Ser/Thr kinases phosphorylate tau protein[15]. Kinases reported to phosphorylate tau on ser262, either directly or indirectly, include calcium/calmodulin dependent protein kinase 2[16,17], protein kinase A[18], microtubule affinity regulating kinase 2[19], phosphorylase kinase[20], and glycogen synthase kinase 3β [21,22]. However, the *in vivo *role of these kinases in the etiology of neurofibrillary tangle formation remains unclear.

We report the use of a high-content siRNA based screening strategy, surveying 572 kinases throughout the human genome, to identify the kinases involved in ser262 tau phosphorylation. After screening validated siRNAs, we identified candidate kinases that either increased or decreased pS262 tau levels. We provide evidence that the eukaryotic translation initiation factor 2 α kinase 2 (EIF2AK2), dual-specificity tyrosine-(Y)-phosphorylation regulated kinase 1A (DYRK1A), and a-kinase anchor protein 13 (AKAP13) proteins contribute to phosphorylation of tau at the 12E8 epitope (pS262/pS356).

## Results

### High-content siRNA screen of the kinome

**Figure 1:**
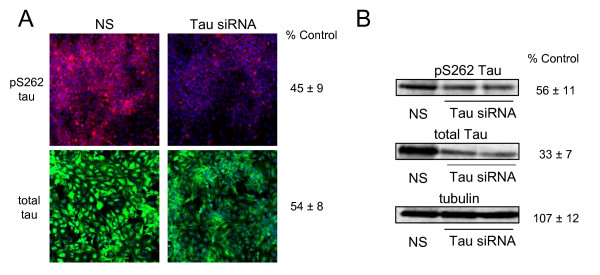
**Quantitative immunofluorescence detection of reductions in 12E8 tau and total tau protein levels using MAPT siRNA**. Shown in A is reduction of both pS262 tau and total tau levels using an siRNA directed at the tau transcript (all splice variants). Using this siRNA, pS262 tau and total tau levels are reduced to 45% and 54% of control values, respectively, following 4 days of transfection with siRNA. pS262 tau is visualized in red. Total tau is in green. Blue staining represents DAPI stained nuclei. Note that significant total tau expression remains following siRNA treatment, likely resulting from the extremely high constitutive levels of tau expression in this cell line. Shown in B is secondary quantitation of the MAPT siRNA effects on 12E8 and total tau using western analyses. Results support the immunofluorescence quantitation shown in A. Representative images are shown. Percent control numbers (± SD) in A represent the average of three independent assays. Westerns in B were performed in triplicate. NS refers to the non-silencing siRNA control.

**Figure 2:**
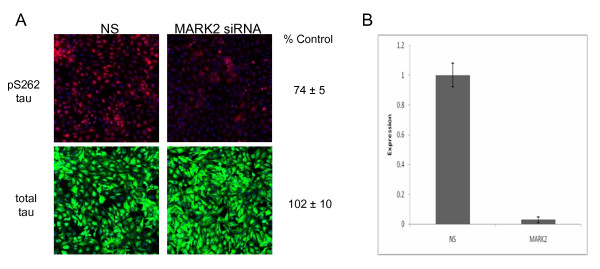
**Quantitative reduction of 12E8 tau with siRNA against MARK2**. Shown in A is detection of pS262 tau and total tau levels following transfection with MARK2 siRNA. Our quantitative assay detects significant reduction of pS262 tau using siRNA directed at the microtubule affinity regulating kinase 2 (MARK2) gene (red panels; p = 0.029 for three replicates). MARK2 siRNA has no effect on the overall levels of total tau protein (green panels), demonstrating that this assay can specifically detect reductions in pS262 tau. Representative images are shown. Percent control numbers represent the average of three independent assays. NS refers to the non-silencing siRNA control. In B, qRT-PCR results confirmed >95% knockdown of MARK2 mRNA relative to non-silencing siRNA control (NS).

To identify the kinases that are important in AD relevant pathologic phosphorylation of tau protein, we developed a cell-based, high-throughput immunofluorescence assay for the rapid detection and quantitation of both total tau and 12E8 tau (pS262 and pS356) protein expression. The assay uses an H4 neuroglioma cell line engineered to overexpress four repeat tau (4R0N). We confirmed that the assay was sensitive to changes in both total tau protein levels and phosphorylated tau protein levels using siRNA directed at the tau (*MAPT*) mRNA (Figure 1A). Importantly, we are able to achieve >95% siRNA transfection efficiency with the H4-tau cell line as assessed by transfection with a lethal siRNA (data not shown). The accuracy of the immunofluorescence quantitation was confirmed by measuring the effects of the *MAPT *siRNA on total and phosphorylated tau protein levels using western analysis (Figure 1B). We next tested if reduced 12E8 tau levels could be detected using siRNA directed against microtubule affinity regulating kinase 2 (*MARK2*), which has previously been shown to phosphorylate tau protein on serine 262[19]. The *MARK2 *siRNA, which reduced *MARK2 *expression >95% at the mRNA level, led to a 26% reduction in 12E8 phosphorylated tau (p = 0.029 for three replicates) and to an insignificant change in total tau protein expression (Figure 2). This result demonstrates that this immunofluorescence assay can detect changes to phosphorylated tau protein independently of significant changes in total tau protein levels.

### siRNA screening results

Following assay validation, we screened the validated kinome siRNA library (Qiagen) for kinases that affect tau phosphorylation status. Cataloging of kinases from the human genome has identified at least 518 kinases[23,24]. This library contains siRNAs to 572 known and predicted kinases. Two siRNAs per target kinase were screened in triplicate for a total of 3,432 target siRNAs screened. Standard paired, two-tailed T-tests were used to determine significant effects relative to non-silencing siRNA controls, which were present in triplicate on each plate. All siRNA screening data are presented as Additional File 1.

To identify potential therapeutic targets that might modify the course of tau phosphorylation and dysfunction in AD, we first identified those kinases that significantly affected the ratio of 12E8 tau to total tau. The ratio of 12E8 tau/total tau could be altered in several ways, either by changes to phosphorylated tau, by changes to total tau, or by a combination of changes to both phosphorylated tau and total tau. For this reason we initially identified siRNAs that significantly reduced 12E8 tau levels with no significant effects on total tau levels relative to non-silencing siRNA controls. Of these kinases, we identified those that significantly affected the ratio of 12E8 tau/total tau relative to the non-silencing siRNA control. These kinases are listed in Table 1 and are candidate kinases acting in specific tau phosphorylation pathways.

**Table 1:** Candidate kinases that phosphorylate 12E8 tau

		**12E8 Tau/Control**			**Total Tau**			**12E8 Tau/Total Tau**		
				
**Gene Name**	**Cell Count**	**Average**	**St Dev**	**p value**	**Average**	**St Dev**	**p value**	**Average**	**St Dev**	**p value**
MARK2	453	0.672	0.044	0.006	0.979	0.039	0.451	0.687	0.060	0.012
PAK3	170	0.803	0.067	0.036	1.112	0.197	0.429	0.730	0.072	0.023
PAK2	287	0.726	0.081	0.028	0.986	0.101	0.831	0.741	0.109	0.054
ADCK5	280	0.716	0.104	0.042	0.934	0.094	0.351	0.765	0.069	0.028
AKAP13	181	0.738	0.069	0.023	0.929	0.070	0.223	0.797	0.086	0.054
LOC55971	152	0.836	0.035	0.014	0.982	0.019	0.241	0.852	0.042	0.026
PLK2	335	0.774	0.087	0.046	0.906	0.114	0.289	0.856	0.052	0.040
DYRK1A	316	0.774	0.051	0.017	0.848	0.104	0.127	0.860	0.038	0.023
MAK	393	1.109	0.021	0.012	0.919	0.037	0.063	1.209	0.054	0.021
ITK	285	1.124	0.036	0.027	0.887	0.059	0.079	1.273	0.118	0.057
PIM1	409	1.248	0.097	0.047	0.965	0.105	0.624	1.297	0.080	0.023
RAGE	397	1.305	0.106	0.038	0.960	0.088	0.511	1.361	0.053	0.007
ITPK1	399	1.324	0.119	0.042	0.930	0.084	0.285	1.424	0.024	0.001
CKB	223	1.537	0.143	0.023	1.065	0.146	0.523	1.454	0.138	0.030
PFKM	375	1.305	0.096	0.032	0.888	0.112	0.227	1.476	0.082	0.010
DGKB	440	1.350	0.096	0.024	0.921	0.163	0.488	1.489	0.200	0.052
SPHK2	333	1.792	0.282	0.040	1.074	0.121	0.399	1.664	0.114	0.010

A list of kinases affecting 12E8 tau phosphorylation. Shown are the most significant effectors of 12E8 tau phosphoylration emerging from a high-content siRNA screen of the complete "kinome." Indicated in the columns from left to right are, the Gene Name, the cell counts (which refers to the number of cells in the well following transfection and is and indicator of potential siRNA toxicity), and then the average fold effect, standard deviation, and p value for 12E8 tau, total tau, and the ratio of 12E8 tau/total tau. For a reference on cell counts, non-siliencing s iRNA control cell counts range from ~330-380, depending on the individual plates in the assay. This tabel contains only those siRNAs that affect pS262 tau/total tau through significant effects on pS262 tau alone. Total tau levels were selected to be insignificantly effected (p > 0.05), although some candidates clearly show a strong trend toward significant reductions in total tau.

**Figure 3:**
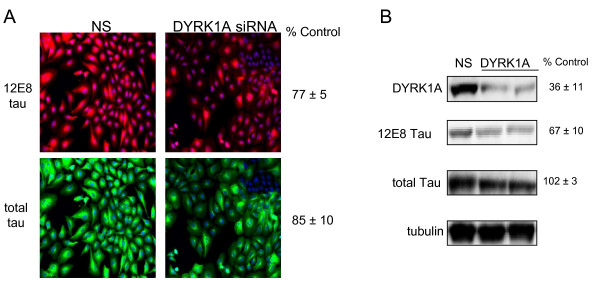
**DYRK1A is required for efficient phosphorylation of 12E8 tau**. Shown in A are results of siRNA screening for DYRK1A siRNA. 12E8 tau is shown in red and total tau is shown in green (see Experimental Methods). For B, H4 cells overexpressing four repeat tau (4R0N) were transfected with siRNA targeting the DYRK1A transcript. Silencing of DYRK1A was confirmed with anti-DYRK1A antibody (top panel). Percent control values represent the average of three independent siRNA transfections and westerns. In A and B, NS refers to the nonsilencing control.

Notably, the microtubule affinity regulating kinase 2 (MARK2) protein, which has been shown to phosphorylate serine 262 of tau protein[19], showed the largest reductions in the ratio of 12E8 tau/total tau when knocked down (69% of control; p = 0.012). This is comparable to the effects seen in our validation assays with MARK2 siRNA, suggesting that the screening assay is performing as expected. Because this kinase is already well known to be a serine 262 tau kinase, we did not confirm this hit via secondary measures. Rather, we chose to validate two additional candidates. These were A-kinase anchor protein 13 (*AKAP13*) and dual specificity tyrosine phosphorylation regulated kinase 1A (*DYRK1A*), which were selected both for the significance of the effect (based on p values for the ratio of 12E8/total tau) and for the known biology of these candidates (see Discussion). The gene encoding DYRK1A is located within the Down Syndrome critical region (DSCR) on chromosome 21. Screening results showed 12E8 tau levels to be reduced to 77 ± 5% of control (p = 0.017), total tau to be 85 ± 10% of control (p = 0.127), which lead to a significant reduction in the ratio of 12E8 tau/total tau to 86 ± 4% of control (p = 0.023; Table 1 and Figure 3A). We confirmed via western analyses that DYRK1A is required for efficient phosphorylation of 12E8 tau. We retransfected our cell line with siRNA targeting *DYRK1A *and quantitated effects on 12E8 tau and total tau levels via western (Figure 3B). Results confirmed that DYRK1A is required for maintaining normal levels of 12E8 tau. The *DYRK1A *siRNA reduced DYRK1A protein expression by over 60%, causing a nearly 40% reduction of 12E8 tau compared to the nonsilencing siRNA control. *DYRK1A *siRNA had no significant effect on total tau levels in this assay. Interestingly, the effects on tau phosphorylation are strikingly larger than observed in the fluorescence screening assay. Reasons for this are unclear, however it suggests that the screening assay may be underestimating the magnitude of effects on tau phosphorylation. Since DYRK1A is a proline- directed serine/threonine kinase and serine 262 is a non-proline directed site on tau, these effects of DYRK1A may result from indirect effects through additional kinases (see Discussion).

**Figure 4:**
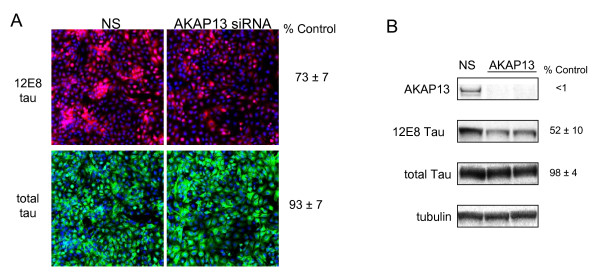
**AKAP13 is required for efficient phosphorylation of 12E8 tau**. Shown in A are results of siRNA screening for AKAP13 siRNA. 12E8 tau is shown in red and total tau is shown in green (see Experimental Methods). For B, H4 cells overexpressing four repeat tau (4R0N) were transfected with siRNA targeting the AKAP13 transcript. Silencing of AKAP13 was confirmed with anti-AKAP13 antibody (top panel). Percent control values represent the average of three independent siRNA transfections and westerns shown. In A and B, NS refers to the non-silencing control.

Tests of *AKAP13 *showed that this protein kinase A (PKA) associated protein is also involved in 12E8 tau phosphorylation. Screening results showed 12E8 tau levels to be reduced to 74 ± 7% (p = 0.023) of control non-silencing siRNA samples, total tau levels to be 93 ± 7% (p = 0.223) of control, leading to a reduction in the ratio of 12E8 tau/total tau to 79 ± 9% (p = 0.054) of control (Table 1 and Figure 4A). Because this phosphorylation profile resembled that of MARK2, a confirmed S262 tau kinase, we proceeded to confirm these effects via secondary measures. Western results demonstrated a role for AKAP13 in maintaining 12E8 tau phosphorylation levels. The *AKAP13 *siRNA reduced AKAP13 protein expression to undetectable levels (Figure 4B), causing a nearly 50% reduction of 12E8 tau. *AKAP13 *siRNA had no significant effect on total tau levels in this assay.

**Figure 5:**
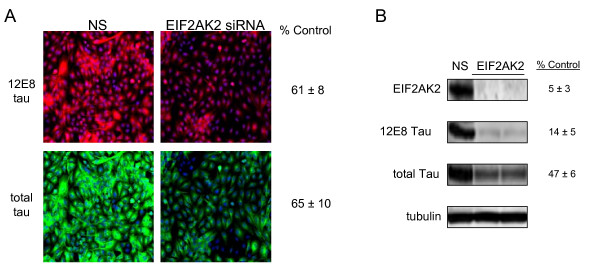
**EIF2AK2 is required for maintenance of normal 12E8 tau levels and total tau expression**. Shown in A is the kinome library screening result for EIF2AK2 siRNA. Effects on 12E8 tau (red) and total tau (green) are shown to the right of each image. In B, triplicate western analyses confirm that EIF2AK2 is required for maintenance of tau and 12E8 tau levels. The *EIF2AK2 *siRNA reduced expression of EIF2AK2 by 95%, resulting in an 85% reduction of 12E8 tau and a greater than 50% reduction of total tau levels. NS is the non-silencing control siRNA.

Some drosophila models have shown that increased tau expression can lead to neurodegeneration, albeit in the absence of NFT pathology[25]. Thus the potential for a tau therapeutic that affects both phosphorylated and total tau levels cannot at this point be diminished. We therefore performed a second analysis of the data to identify those kinases that significantly affected both 12E8 and total tau levels simultaneously. To identify those candidates, we pulled out kinases that had statistically significant effects on both total tau levels and 12E8 tau levels relative to controls, irrespective of the resulting effects on the ratio of 12E8 tau/total tau. These are presented in Table 2. Interestingly, two siRNAs to the eukaryotic translation initiation factor 2 α kinase 2 (*EIF2AK2*) gene significantly reduced 12E8 and total tau levels (Figure 5 and Table 2). These siRNAs reduced 12E8 tau levels to 61 ± 8% (p = 0.012) and 72 ± 7% (p = 0.018) of non-silencing siRNA controls and reduced total tau levels to 65 ± 10% (p = 0.026) and 71 ± 8% (p = 0.025) of control values, respectively. The comparable eductions of both phosphorylated and total tau levels led to no change in the ratio of 12E8 tau/total tau.

**Table 2:** Candidate kinases affecting total tau and 12E8 tau expression

		**12EB Tau/Control**			**Total Tau**			**12E8 Tau/Total Tau**		
				
**Gene Name**	**Cell Count**	**Average**	**St Dev**	**p value**	**Average**	**St Dev**	**p value**	**Average**	**St Dev**	**p value**
EIF2AK2	217	0.606	0.075	0.012	0.647	0.102	0.026	0.962	0.237	0.806
EIF2AK2	318	0.719	0.069	0.018	0.706	0.082	0.025	1.038	0.257	0.822
CDKL1	398	0.852	0.032	0.015	0.761	0.085	0.040	1.132	0.157	0.281
DCK	363	1.068	0.015	0.017	0.701	0.051	0.010	1.530	0.107	0.013
DGKQ	406	1.119	0.042	0.039	0.805	0.059	0.029	1.394	0.111	0.026
PFKFB3	458	1.204	0.046	0.016	0.813	0.029	0.008	1.483	0.088	0.011
ERK8	466	1.234	0.063	0.024	0.807	0.009	0.001	1.528	0.084	0.008
STK19	347	1.334	0.103	0.030	0.654	0.115	0.035	2.065	0.231	0.015
PRKG2	413	1.384	0.152	0.048	0.721	0.107	0.046	1.959	0.412	0.056
MAP2K1IP1	436	1.393	0.052	0.006	0.831	0.048	0.026	1.679	0.123	0.011

Kinases that affect bioth 12E8 tau and total tau levels. Shown are those kinases that affect total tau levels and 12E8 tau levels, irrespective of significant effects to the ratio of 12E8 tau/total tau. Columns are labeled as in Table 1.

To confirm these screening results for *EIF2AK2*, we retransfected our cell line with *EIF2AK2 *siRNA. Western blotting results confirmed that EIF2AK2 is required for maintaining normal levels of 12E8 tau and total tau protein. *EIF2AK2 *siRNA treatment reduced EIF2AK2 levels to 5% of control non-silencing siRNA, leading to a reduction of 12E8 tau levels to 14% of control non-silencing siRNA (Figure 5B). Total tau levels were reduced to 47% of control values. Again, this effect is strikingly larger than revealed in the immunofluorescence screening assay, providing additional confirmation that the assay is likely to be underestimating the true magnitude of the effects of some gene candidates.

## Discussion

### High-throughput, high-content siRNA screening in neurodegenerative diseases

We report the combination of a high-throughput survey of the entire kinome with a new assay approach to understand a critical component of tau pathology, and provide new targets for the discovery of tau-modifying AD treatments. We have developed a sensitive and specific assay for the detection of hyperphosphorylated tau protein on an AD-relevant site. The assay is not only amenable to additional tau phosphorylation sites, but also to those identifiable cell-based endpoints related to other neurodegenerative diseases. Imaging technology has only recently become available to enable these high-content analyses. However, with a persistent need for the identification of novel drug targets to treat increasingly common neurodegenerative diseases, RNA interference screening technology now provides a promising approach.

### EIF2AK2 and DYRK1A as important mediators of AD-related tau hyperphosphorylation

The cell line used in these studies overexpresses 4R0N tau protein. This is technically necessary to achieve levels of phosphorylated tau protein sufficient enough for detection of modulation of phosphorylation status. Because of this overexpression there is the possibility of creating a significant cytoplasmic pool of tau protein that is not bound to microtubules. This raises the possibility that some phospho-epitopes that are normally protected (via microtubule binding or interactions with other proteins at physiologic concentrations) may be exposed and phosphorylation pathways that do not normally regulate tau would have an effect in this assay. However, the identification of MARK2 as a significant pS262 tau phosphorylator (Table 1) suggests that tau regulatory pathways are functioning on the 12E8 epitope in this cell line. Nevertheless, the true applicability of the kinases that we identify here will emerge only after subsequent follow up studies in neuronal cell lines and *in vivo *in animal models of AD and of tauopathies.

Both the eukaryotic translation initiation factor 2 α kinase 2 gene (*EIF2AK2*) and the dual specificity tyrosine phosphorylation regulated kinase 1A gene (*DYRK1A*) have been implicated previously in neurodegeneration in AD. Polymorphisms within the eukaryotic translation initiation factor 2 α kinase 2 (EIF2AK2) gene have been genetically associated with AD[26]. In addition, EIF2AK2 has been shown to be activated in AD brain[27,28] and has been implicated in neuronal apoptosis resulting from toxic β-amyloid peptides[28]. EIF2AK2 has also been implicated in the extrastriatal neurodegeneration of Parkinson's disease and Huntington's disease[29]. In our study, two siRNAs that reduced EIF2AK2 protein expression by 95% caused a significant reduction of pS262 tau levels and a lesser but significant reduction in total tau levels (Table 2). These findings suggest an important role for EIF2AK2 in the expression of total and 12E8 phosphorylated tau protein. Moreover, the comparable effects seen for total tau and 12E8 tau suggest the possibility that EIF2AK2 may be required for the maintenance of multiple forms of phosphorylated tau, in addition to 12E8 tau. Deciphering the mechanisms through which this regulation occurs will require additional experimentation.

Polymorphisms within the DYRK1A locus also have been associated with AD in some AD patient populations[30]. Moreover, DYRK1A has been implicated in phosphorylation of tau protein on threonine 212, serine 202, and serine 404[31,32]. In this work we demonstrate a role for DYRK1A in the phosphorylation of tau protein on the 12E8 epitope, independent of significant effects on total tau protein expression. Interestingly, DYRK1A is a proline- directed serine/threonine kinase. Serine 262 is not a proline-directed phosphorylation site on tau. One possible scenario is that DYRK1A effects on S262 could occur through activities of additional kinases downstream of DYRK1A function. Presumably, silencing of those kinases would also affect 12E8 tau levels in our assay and strong candidates for downstream kinases through which DYRK1A may be signaling are those additional kinases in Table 1. Ongoing work is directed at identifying the additional signaling components involved in mediating the effects of DYRK1A on 12E8 tau phosphorylation and at determining if DYRK1A is capable of directly phosphorylating tau protein on serine 262/serine 356.

### A possible role for AKAP13 in the regulation of tau phosphorylation

Although it is clear that AKAP13 has significant effects on 12E8 tau levels in our cell line, the mechanisms through which this protein may affect tau are unclear. The AKAP13 protein is an ~320 kD protein that functions as an anchor protein for the regulatory subunit of protein kinase A, effectively localizing the PKA holoenzyme to discrete locations within the cell. Protein kinase A has been reported to increase tau phosphorylation *in vitro *on the 12E8 epitope under some conditions, such as in response to the binding of α-synuclein to tau protein[33]. Additionally, AKAP13, and other members of the AKAP family, have a Dbl homology (DH) domain that functions as a guanine nucleotide exchange activation domain for the Rho/Rac family of GTP binding proteins. Thus, AKAP13 coordinates a Rho signaling pathway that ultimately leads to cytoskeletal reorganization. In addition, other AKAP family members have been implicated in regulating synaptic plasticity and long-term memory formation[34,35]. These combined observations suggest interesting possibilities wherein AKAP13 could anchor a signaling complex at the cell membrane that ultimately regulates tau phosphorylation and microtubule dynamics in response to extracellular stimuli. However, direct connections to tau phosphorylation based on the currently known functions of AKAP13 are unclear but certainly worthy of further study.

## Conclusions

One additional implication of our findings is that phosphorylation of the 12E8 epitope is likely controlled by multiple redundant signaling pathways. This conclusion is supported by the fact that silencing of no single kinase fully blocked 12E8 tau phosphorylation. In addition, the complexity of these signaling pathways is also evident in the observation that knockdown of multiple kinases leads to significant increases in 12E8 tau levels (Table 1). When coupled with recent findings showing that phosphorylation of tau at multiple sites appears to be required for neurotoxicity of tau[14], it is becoming clear that the regulatory pathways controlling cellular tau function and dysfunction in disease are complex. Our results were generated in a cell line that overexpresses four repeat tau protein, a non-physiological condition. However, if in subsequent experiments these results extrapolate to mammalian brain, it would suggest that a therapeutic strategy to decrease tau hyperphosphorylation in AD and other tauopathies may require the simultaneous modulation of several phosphorylation sites. This is one reason why candidate targets that reduce total tau levels, such as EIF2AK2, are appealing since they are likely to simultaneously affect multiple phosphorylation sites. Untangling the complex regulatory networks of tau hyperphosphorylation is a daunting task. However, our results provide an important first step in defining the scope of kinases and associated proteins that may be involved in phosphorylating tau on a site highly relevant to tau dysfunction, tau pathology, and AD. Further work remains to determine the complex pathways through which the kinases identified here may interact to control tau phosphorylation levels.

## Methods

### Generation of 4R0N tau overexpressing H4 cell line

The Human H4 neuroglioma cell line (ATCC) was transfected with a 4R0N tau construct in pcDNA3.1. Following positive selection using geneticin (Invitrogen), individual clones were isolated and screened for tau expression using immunocytochemistry with a rabbit anti-tau antibody (Dako). A single stable tau overexpressing cell line was selected for subsequent siRNA studies. Cells were maintained in Dulbecco's Modified Eagle Medium (Invitrogen) supplemented with 10% fetal bovine serum (Invitrogen), 1% penicillin-streptomycin, geneticin (0.25 mg/ml), and 2 mM L-Glutamine (Invitrogen). Cells were split 1:10 at 90% confluency, twice a week.

### High Throughput siRNA Kinome Screen

#### Preparation of siRNA library plates

Chemically synthesized siRNA (18.6 μg) from Qiagen's validated human kinase siRNA Set 2.0 were printed into 96-well black clear bottom plates (Corning #3904) using a BioMek FX (Beckman Coulter). Printed plates were then foil sealed and stored at -80°C until use.

#### High Content siRNA Screening Assay

In order to identify genes involved in modulation of tau phosphorylation, we developed a high content siRNA screening assay. Briefly, 4R0N tau overexpressing H4 cells were reverse-transfected with library siRNA and siLentfect (Bio-Rad) using semi-automated transfection as follows: Screening plates were thawed and siRNA was complexed by addition of 50 μl of diluted siLentfect in OptiMEM (Invitrogen) using a μFill (BioTek) followed by incubation for thirty minutes at RT. Cells were trypsinized and resuspended in growth media without penicillin-streptomycin and added at a concentration of 3000 cell/well in 50 μl using a μFill followed by incubation for 5 minutes at room temperature (RT). Assay plates were incubated at 37°C and 5% CO_2_, for 96 hours.

Plates were washed twice with 1× Tris-buffered saline (TBS) (Fisher) and fixed for 15 minutes with4% paraformaldehyde (PFA) at RT. Plates were then washed twice with 1× TBS and incubated with blocking buffer (TBS with 5% normal goad serum, 0.2% sodium azide, 1% bovine serum albumin (BSA) and 0.1% NP-40) for 1 hour at RT. Fixed cells were next incubated with 1 μg/ml of phospho-tau antibody 12E8 (Elan Pharmaceuticals) at 4°C overnight. Plates were washed three times with 1× TBS-T (TBS with 0.1% Tween-20) followed by incubation with 1:200 dilution of rabbit anti-human tau (Dako) for 1 hr at RT. Plates were then washed twice with 1× TBS-T followed by incubation with a secondary antibody cocktail of 2 μg/ml FITC-Goat anti-rabbit IgG, 2 μg/ml Cy5- Goat anti-mouse IgG (Jackson Immunore search) and 10 μg/ml Hoechst 33342 (Invitrogen) in blocking buffer for 30 minutes at RT. Plates were then washed once with TBS-T followed by twice with TBS leaving the last wash in the plates. Plates were stored at 4°C overnight prior to analysis. Plates were imaged and then analyzed on the INCell 3000 (GE Healthcare). The analysis module (Object Intensity 01) identified each cell by the blue staining of the nuclei, and measured both red (Cy5) and green (FITC) intensities in a 4 pixel ring around the nuclei.

#### Western Blotting

4R0N tau overexpressing H4 cells were reverse-transfected with siLentfect complexed with target siRNA in 6 well plates. Cells were grown for 72 hours at 37°C, 5% CO_2_. Cell lysates were prepared using the Complete Lysis-M, EDTA-free kit (Roche Applied Science) and quantitated using the BCA protein assay (Pierce). Protein from lysates (20 μg) were separated by SDS-PAGE and transferred to nitrocellulose. Membrane was blocked in 5% BSA for one hour at RT. Membranes were probed with primary antibody overnight at 4°C on a rocker. Membranes were subsequently washed with TBS-T and probed with secondary antibody 1:25000 dilution of HRP-GAM (Jackson Immunoresearch) for forty-five minutes. Membranes were further washed and developed with Super Signal West Femto Maximum Sensitivity Substrate Kit (Promega) and imaged. To test multiple primary antibodies, membranes were stripped for 15 minutes at RT using ReBlot Plus Mild Antibody Stripping Solution (Millipore). Membranes were then washed again for 5 minutes at RT then blocked for one hour in 5% BSA. Membrane was reprobed overnight at 4°C with an anti-Tubulin antibody (1:25000; ICN Biomedicals, Inc.). Antibodies used for detection included anti-tau (1:1000; Dako) primary antibody, 12E8 antibody (1:7500), anti-EIF2AK2 (1:1000; Abcam), anti-DYRK1A (1:500; Abcam), and anti-AKAP13 (1:2000; Bethyl Laboratories, Inc).

## Abbreviations

All are defined in the text upon first use.

## Authors' contributions

All authors have read and approved the final manuscript. DOA, RR (developed the siRNA screrening assay and led and performed target validation efforts); GRB, CB, GDB, DRH, JAH, KMB, LG, AG, JR (assisted with siRNA assay development); CD(helped generate the H4-tau cell line); DF, BM (assisted with kinase validation experiments); EMR, Hutton MH, DAS, SM (provided critical review of manuscript and helped with interpretation of results); TD (Led the development of the siRNA screening assay, the interpretation of results, the selection of kinases for validation, and wrote the manuscript). All authors read and approved the final manuscript.

## Supplementary Material

Additional file 1 **Total tau and 12E8 tau data for all siRNAs in the Qiagen Kinome siRNA library**. All data from the kinome siRNA library screen. p Tau refers to 12E8 tau. Cumulative results columns represent the average of the three independent experiments reported. Click here for file

## References

[B1] Seabrook GR Ray WJ Shearman M Hutton M Beyond amyloid: the next generation of Alzheimer's disease therapeutics Mol Interv 2007 7 5 261 270 10.1124/mi.7.5.8 17932415

[B2] Kidd M Paired helical filaments in electron microscopy of Alzheimer's Disease Nature 1963 197 192 193 10.1038/197192b0 14032480

[B3] Terry R The fine structure of neurofibrillary tangles in Alzheimer's disease J Neuropathol Exp Neuro 1963 22 629 642 10.1097/00005072-196310000-00005 14069842

[B4] Grundke-Iqbal I Iqbal K Tung YC Quinlan M Wisniewski HM Binder LI Abnormal phosphorylation of the microtubule-associated protein tau (tau) in Alzheimer cytoskeletal pathology Proc Natl Acad Sci USA 1986 83 13 4913 4917 10.1073/pnas.83.13.4913 3088567 PMC323854

[B5] Gustke N Steiner B Mandelkow EM Biernat J Meyer HE Goedert M Mandelkow E The Alzheimer-like phosphorylation of tau protein reduces microtubule binding and involves Ser-Pro and Thr-Pro motifs FEBS Lett 1992 307 2 199 205 10.1016/0014-5793(92)80767-B 1644173

[B6] Ihara Y Nukina N Miura R Ogawara M Phosphorylated tau protein is integrated into paired helical filaments in Alzheimer's disease J Biochem 1986 99 6 1807 1810 2427509 10.1093/oxfordjournals.jbchem.a135662

[B7] Kosik KS Orecchio LD Bakalis S Neve RL Developmentally regulated expression of specific tau sequences Neuron 1989 2 4 1389 1397 10.1016/0896-6273(89)90077-9 2560640

[B8] Lace GL Wharton SB Ince PG A brief history of tau: the evolving view of the microtubule-associated protein tau in neurodegenerative diseases Clin Neuropathol 2007 26 2 43 58 17416103 10.5414/npp26043

[B9] Johnson GV Bailey CD Tau, where are we now? J Alzheimers Dis 2002 4 5 375 398 12446970 10.3233/jad-2002-4505

[B10] Trinczek B Biernat J Baumann K Mandelkow EM Mandelkow E Domains of tau protein, differential phosphorylation, and dynamic instability of microtubules Mol Biol Cell 1995 6 12 1887 1902 8590813 10.1091/mbc.6.12.1887 PMC366657

[B11] Augustinack JC Schneider A Mandelkow EM Hyman BT Specific tau phosphorylation sites correlate with severity of neuronal cytopathology in Alzheimer's disease Acta Neuropathol 2002 103 1 26 35 10.1007/s004010100423 11837744

[B12] Mi K Johnson GV The role of tau phosphorylation in the pathogenesis of Alzheimer's disease Curr Alzheimer Res 2006 3 5 449 463 10.2174/156720506779025279 17168644

[B13] Sengupta A Kabat J Novak M Wu Q Grundke-Iqbal I Iqbal K Phosphorylation of tau at both Thr 231 and Ser 262 is required for maximal inhibition of its binding to microtubules Arch Biochem Biophys 1998 357 2 299 309 10.1006/abbi.1998.0813 9735171

[B14] Steinhilb ML Dias-Santagata D Fulga TA Felch DL Feany MB Tau phosphorylation sites work in concert to promote neurotoxicity in vivo Mol Biol Cell 2007 18 12 5060 5068 10.1091/mbc.E07-04-0327 17928404 PMC2096612

[B15] Johnson GV Hartigan JA Tau protein in normal and Alzheimer's disease brain: an update J Alzheimers Dis 1999 1 4-5 329 351 12214129 10.3233/jad-1999-14-512

[B16] Yamamoto H Yamauchi E Taniguchi H Ono T Miyamoto E Phosphorylation of microtubule-associated protein tau by Ca2+/calmodulin-dependent protein kinase II in its tubulin binding sites Arch Biochem Biophys 2002 408 2 255 262 10.1016/S0003-9861(02)00556-8 12464279

[B17] Singh TJ Wang JZ Novak M Kontzekova E Grundke-Iqbal I Iqbal K Calcium/calmodulin-dependent protein kinase II phosphorylates tau at Ser-262 but only partially inhibits its binding to microtubules FEBS Lett 1996 387 2-3 145 148 10.1016/0014-5793(96)00485-1 8674537

[B18] Litersky JM Johnson GV Jakes R Goedert M Lee M Seubert P Tau protein is phosphorylated by cyclic AMP-dependent protein kinase and calcium/calmodulin-dependent protein kinase II within its microtubule-binding domains at Ser-262 and Ser-356 Biochem J 1996 316 Pt 2 655 660 8687413 10.1042/bj3160655 PMC1217397

[B19] Drewes G Trinczek B Illenberger S Biernat J Schmitt-Ulms G Meyer HE Mandelkow EM Mandelkow E Microtubule-associated protein/microtubule affinity-regulating kinase (p110mark). A novel protein kinase that regulates taumicrotubule interactions and dynamic instability by phosphorylation at the Alzheimer-specific site serine 262 J Biol Chem 1995 270 13 7679 7688 10.1074/jbc.270.13.7679 7706316

[B20] Paudel HK The regulatory Ser262 of microtubule-associated protein tau is phosphorylated by phosphorylase kinase J Biol Chem 1997 272 3 1777 1785 8999860

[B21] Kosuga S Tashiro E Kajioka T Ueki M Shimizu Y Imoto M GSK-3beta directly phosphorylates and activates MARK2/PAR-1 J Biol Chem 2005 280 52 42715 42722 10.1074/jbc.M507941200 16257959

[B22] Song JS Yang SD Tau protein kinase I/GSK-3 beta/kinase FA in heparin phosphorylates tau on Ser199, Thr231, Ser235, Ser262, Ser369, and Ser400 sites phosphorylated in Alzheimer disease brain J Protein Chem 1995 14 2 95 105 10.1007/BF01888367 7786411

[B23] Manning G Whyte DB Martinez R Hunter T Sudarsanam S The protein kinase complement of the human genome Science 2002 298 5600 1912 1934 10.1126/science.1075762 12471243

[B24] Johnson SA Hunter T Kinomics: methods for deciphering the kinome Nat Methods 2005 2 1 17 25 10.1038/nmeth731 15789031

[B25] Wittmann CW Wszolek MF Shulman JM Salvaterra PM Lewis J Hutton M Feany MB Tauopathy in Drosophila: neurodegeneration without neurofibrillary tangles Science 2001 293 5530 711 714 10.1126/science.1062382 11408621

[B26] Bullido MJ Martinez-Garcia A Tenorio R Sastre I Munoz DG Frank A Valdivieso F Double stranded RNA activated EIF2 alpha kinase (EIF2AK2; PKR) is associated with Alzheimer's disease Neurobiol Aging 2007 10.1016/j.neurobiolaging.2007.02.023 17420072

[B27] Peel AL Bredesen DE Activation of the cell stress kinase PKR in Alzheimer's disease and human amyloid precursor protein transgenic mice Neurobiol Dis 2003 14 1 52 62 10.1016/S0969-9961(03)00086-X 13678666

[B28] Chang RC Wong AK Ng HK Hugon J Phosphorylation of eukaryotic initiation factor-2alpha (eIF2alpha) is associated with neuronal degeneration in Alzheimer's disease Neuroreport 2002 13 18 2429 2432 10.1097/00001756-200212200-00011 12499843

[B29] Bando Y Onuki R Katayama T Manabe T Kudo T Taira K Tohyama M Double-strand RNA dependent protein kinase (PKR) is involved in the extrastriatal degeneration in Parkinson's disease and Huntington's disease Neurochem Int 2005 46 1 11 18 10.1016/j.neuint.2004.07.005 15567511

[B30] Kimura R Kamino K Yamamoto M Nuripa A Kida T Kazui H Hashimoto R Tanaka T Kudo T Yamagata H The DYRK1A gene, encoded in chromosome 21 Down syndrome critical region, bridges between beta-amyloid production and tau phosphorylation in Alzheimer disease Hum Mol Genet 2007 16 1 15 23 10.1093/hmg/ddl437 17135279

[B31] Ryoo SR Jeong HK Radnaabazar C Yoo JJ Cho HJ Lee HW Kim IS Cheon YH Ahn YS Chung SH DYRK1A-mediated hyperphosphorylation of Tau. A functional link between Down syndrome and Alzheimer disease J Biol Chem 2007 282 48 34850 34857 10.1074/jbc.M707358200 17906291

[B32] Woods YL Cohen P Becker W Jakes R Goedert M Wang X Proud CG The kinase DYRK phosphorylates protein-synthesis initiation factor eIF2Bepsilon at Ser539 and the microtubule-associated protein tau at Thr212: potential role for DYRK as a glycogen synthase kinase 3-priming kinase Biochem J 2001 355 Pt 3 609 615 11311121 10.1042/bj3550609 PMC1221774

[B33] Jensen PH Hager H Nielsen MS Hojrup P Gliemann J Jakes R alphasynuclein binds to Tau and stimulates the protein kinase A-catalyzed tau phosphorylation of serine residues 262 and 356 J Biol Chem 1999 274 36 25481 25489 10.1074/jbc.274.36.25481 10464279

[B34] Dell'Acqua ML Smith KE Gorski JA Horne EA Gibson ES Gomez LL Regulation of neuronal PKA signaling through AKAP targeting dynamics Eur J Cell Biol 2006 85 7 627 633 10.1016/j.ejcb.2006.01.010 16504338

[B35] Bauman AL Goehring AS Scott JD Orchestration of synaptic plasticity through AKAP signaling complexes Neuropharmacology 2004 46 3 299 310 10.1016/j.neuropharm.2003.09.016 14975685

